# Predicting the individualized risk of human immunodeficiency virus infection among sexually active women in Ethiopia using a nomogram: prediction model development and validation

**DOI:** 10.3389/fpubh.2024.1375270

**Published:** 2024-06-24

**Authors:** Tigabu Kidie Tesfie, Tirualem Zeleke Yehuala, Muluken Chanie Agimas, Getaneh Awoke Yismaw, Sisay Maru Wubante, Bezawit Melak Fente, Nebiyu Mekonnen Derseh

**Affiliations:** ^1^Department of Epidemiology and Biostatistics, Institute of Public Health, College of Medicine and Health Sciences, University of Gondar, Gondar, Ethiopia; ^2^Department of Health Informatics, Institute of Public Health, College of Medicine and Health Sciences, University of Gondar, Gondar, Ethiopia; ^3^Department of General Midwifery, School of Midwifery, College of Medicine and Health Sciences, University of Gondar, Gondar, Ethiopia

**Keywords:** HIV, sexually active women, prediction model, Ethiopia, nomogram

## Abstract

**Introduction:**

Women are more vulnerable to HIV infection due to biological and socioeconomic reasons. Developing a predictive model for these vulnerable populations to estimate individualized risk for HIV infection is relevant for targeted preventive interventions. The objective of the study was to develop and validate a risk prediction model that allows easy estimations of HIV infection risk among sexually active women in Ethiopia.

**Methods:**

Data from the 2016 Ethiopian Demographic and Health Survey, which comprised 10,253 representative sexually active women, were used for model development. Variables were selected using the least absolute shrinkage and selection operator (LASSO). Variables selected by LASSO were incorporated into the multivariable mixed-effect logistic regression model. Based on the multivariable model, an easy-to-use nomogram was developed to facilitate its applicability. The performance of the nomogram was evaluated using discrimination and calibration abilities, Brier score, sensitivity, and specificity. Internal validation was carried out using the bootstrapping method.

**Results:**

The model selected seven predictors of HIV infection, namely, age, education, marital status, sex of the household head, age at first sex, multiple sexual partners during their lifetime, and residence. The nomogram had a discriminatory power of 89.7% (95% CI: 88.0, 91.5) and a calibration *p*-value of 0.536. In addition, the sensitivity and specificity of the nomogram were 74.1% (95% CI: 68.4, 79.2) and 80.9% (95% CI: 80.2, 81.7), respectively. The internally validated model had a discriminatory ability of 89.4% (95% CI: 87.7, 91.1) and a calibration p-value of 0.195. Sensitivity and specificity after validation were 72.9% (95% CI: 67.2, 78.2) and 80.1% (95% CI: 79.3, 80.9), respectively.

**Conclusion:**

A new prediction model that quantifies the individualized risk of HIV infection has been developed in the form of a nomogram and internally validated. It has very good discriminatory power and good calibration ability. This model can facilitate the identification of sexually active women at high risk of HIV infection for targeted preventive measures.

## Introduction

By the end of 2022, the number of people living with human immunodeficiency virus/acquired immunodeficiency syndrome (HIV/AIDS) worldwide was 39 million. The World Health Organization’s (WHO) African region remains most severely affected, with approximately 1 in every 25 adults living with HIV and accounting for two-thirds of the global morbidity burden (1). This makes HIV/AIDS one of the most serious worldwide public health problems (2). Sub-Saharan Africa (SSA) is home to only 12% of the global population, yet it accounts for 71% of the global burden of HIV infection (3). Women are disproportionately affected by HIV transmission, accounting for 54% of the total number of people living with HIV (4). According to the Joint United Nations Programme on HIV/AIDS (UNAIDS) global report of 2022, the southern and eastern parts of Africa remain the most heavily affected region by HIV, with 20.6 million (54% of the global burden) people living with HIV (5).

The global HIV/AIDS burden reflects the continued transmission of HIV as a public health problem despite reductions in incidence and expanded access to antiretroviral treatment, which have helped to reduce HIV-related deaths (1). Accelerated reductions of new HIV infections are needed to end AIDS as a public health threat by 2030 (5). The world’s continued commitment toward HIV/AIDS response and “ending AIDS” as a public health threat by 2030 can be signaled by the 95–95-95 targets (6). This strategy calls for 95% of people living with HIV to know their status, 95% of people with HIV diagnoses to receive antiretroviral therapy (ART), and 95% of people receiving ART to have viral suppression by 2025 (7).

The burden of HIV/AIDS remains high in Ethiopia, even though a considerable scale-up of comprehensive HIV/AIDS interventions has been made so far. According to the 2016 Global Burden of Disease study, Ethiopia achieved a 77 and 0.4% reduction between 1990 and 2016 in the incidence of infection and mortality, respectively. Ethiopia achieved the Millennium Development Goals (MDGs) toward HIV/AIDS, which aimed to reduce the incidence by 50% from 2000 to 2015 (8). In 2022, the Ethiopian Public Health Institute (EPHI) report revealed that approximately 573,538 adults were living with HIV, which makes the national adult HIV prevalence of 0.91% (9). This shows the presence of high infection transmission that poses a threat to target achievements. To achieve HIV/AIDS-related Sustainable Development Goals in Ethiopia, concerted efforts are required for comprehensive HIV/AIDS interventions (8).

HIV/AIDS is a threat to socioeconomic development beyond morbidity and mortality. It is affecting educational achievements, productivity and economic growth, social status, and other components of human development, including human rights and gender equality (10–12). Hence, HIV/AIDS demands a greater degree of well-thought-out, intensive, and coordinated action by sub-national, national, and international agencies (13, 14). HIV/AIDS in women has a strong implication for national and global socioeconomic development since women play a crucial role in development activities such as trade, agriculture, and family support (15).

According to the studies conducted in different countries across Africa, several factors were found to be significantly associated with HIV, infection including age (16–18), marital status (16, 18, 19), educational attainment (17), wealth index (20), sex of the household head (21), residence (17, 18), region (17), number of sexual partners (17, 18), age at first sex (22), and sexually transmitted infections (19). However, none of these studies apply predictive modeling techniques to assess which combination of variables best predicts HIV infection risk.

Unprotected heterosexual encounters are the primary mechanism for HIV transmission in Africa. Hence, consistent condom use remains the most effective method of HIV prevention. However, according to studies conducted among Mozambican women, consistent condom use depends on sociodemographic characteristics that include educational status, marital status, and age. In addition, psychosocial factors such as perception of barriers to safe sex, condom use negotiation self-efficacy, and HIV prevention knowledge play a significant role in the utilization of condoms (23, 24). In addition, three mechanisms, namely, pre-exposure prophylaxis, behavioral change communications, and early initiation of ART remain vital strategies for preventing HIV/AIDS transmission (25).

Identification of individuals at high risk for HIV/AIDS and linking them to prevention services is essential for continued progress toward ending HIV as a public health threat (26, 27). Risk estimation tools, such as nomograms, could play a role in directing targeted preventive strategies through the quantification of individualized risk (25). Predicting the risk of HIV infection is also used as an early-warning system to notify prevention programs (28). Women are biologically more prone to HIV infection for the reason that a greater mucus area is exposed to the virus during sexual intercourse. Their higher risk is also linked to gender inequalities and the economic and social pressures of poverty, which intensify HIV infection risk (29). Developing a risk prediction model for these highly vulnerable groups of the population had a vital role in the prevention of HIV infection. Prediction models can help clinicians, public health professionals, and clients by directing the decision-making process in the choice of possible interventions through individualized risk stratification with the hope of improving patient outcomes and quality of care (30, 31).

Unlike previous studies conducted among sexually active women (16–21) that primarily focused on investigating and identifying statistically significant associations between factors and HIV status, we utilize a predictive modeling technique to identify a combination of factors that best predicts the individualized risk of HIV infection among sexually active women, develop user-friendly nomogram based on the best combination of variables, evaluate the extent to which the nomogram can differentiate between negative and positive HIV status, assess the agreement between predicted probability and actual probability of HIV infection, and assess the internal validity of the predictive model developed for individualized HIV infection risk estimation. Therefore, this study aims to develop and validate a nomogram for an individualized HIV infection risk estimation among sexually active women in Ethiopia using nationally representative data.

## Methods

### Study design, setting, and period

A nationwide, cross-sectional Demographic and Health Survey (DHS) was conducted in Ethiopia between 18 January and 27 June 2016 (32).

### Sampling procedure, population, and data sources

Participants in the 2016 Ethiopian Demographic and Health Survey (EDHS) were identified using a two-stage stratified cluster sampling technique. In the first stage of the sampling, 645 clusters (202 urban and 443 rural) were selected based on the 2007 Ethiopian Population and Housing Census sampling frame. In the second stage, a fixed number of 28 households per cluster were randomly selected from the household list. The source population consisted of all sexually active women in Ethiopia, whereas the study population was all sexually active women in the selected enumeration areas. A total of 15,683 women of reproductive age were interviewed. Out of these, 3,721 women had never had sex. From a total of 11,962 women who read the consent statement, 11,185 (a weighted sample of 10,253) women granted permission for HIV testing. A total weighted sample of 10,253 sexually active women were included in this study. Details of the survey methodology have been published elsewhere (32). The data used for this study were obtained from the DHS program using the link http://www.dhsprogram.com.

### Variables of the study

The outcome variable was HIV status (positive or negative). To confirm the diagnosis, interviewers collected blood specimens by finger pricking from sexually active women who granted consent for HIV testing. Blood samples were dried overnight, packed the following morning for storage, and then transported to the EPHI laboratory. Until further testing, blood samples were stored at −20°C. The HIV testing algorithm used to determine HIV status has been published elsewhere (32). The predictor variables included age, education, marital status, household wealth index, sex of the household head, residence, region, age at first sex, multiple sexual partners during the lifetime, multiple sexual partners in 1 year, diagnosed STIs, genital ulcers, and genital discharge (16–21). For all predictor variables, ascertainments were made based on women’s responses to the interviewer-administered questionnaire.

Regarding categorization, predictor variables were categorized as follows: age (15-19, 20-24, 25-29, 30-34, 35-39, 40-44, and 45-49), education (no, primary, secondary, and higher), marital status (single, married/cohabiting, and separated/widowed/divorced), wealth index (poorest, poorer, middle, richer, and richest), sex of the household head (male or female), residence (urban or rural), region (Tigray, Afar, Amhara, Oromia, Somali, Benishangul-Gumuz, South Nation Nationalities and Peoples’ Region (SNNPR), Gambela, Harari, Addis Ababa, and Dire Dawa), age at first sex in years (≤ 14, 15–17, and ≥ 18), multiple sexual partners during lifetime (no or yes), multiple sexual partners in 1 year (no or yes), diagnosed STIs (no or yes), genital ulcers (no or yes), and genital discharge (no or yes).

### Data processing and analysis

Data obtained from the DHS program were imported into Stata version 17 software. The individual and HIV datasets were merged using the cluster number (v001), household number (v002), and respondent’s line number (v003). Samples were weighted using the weight variable (hiv05) to maintain representativeness. Missing data were managed according to the DHS guidelines (33). The results are presented in the form of text, tables, and figures.

During the model development process, variables were screened for chi-square assumptions. Hence, variables that fulfilled the chi-square assumptions were entered into the least absolute shrinkage and selection operator (LASSO) regression model. Then, variables with non-zero coefficients from the LASSO model were used in the multivariable analysis. DHS data have a clustering nature, in which women from the same cluster are more dependent than women from different clusters. We used the intra-class correlation coefficient (ICC) to estimate the clustering effect. The ICC was greater than 10%, indicating that considering the clustering effect is mandatory to obtain appropriate estimates of association for the development of the prediction model. Therefore, the multivariable mixed-effects binary logistic regression model was fitted. Insignificant variables were reduced one by one using the likelihood ratio test. Based on the predictors retained in the reduced model, an individualized HIV infection risk estimation tool was developed in the form of a nomogram.

The nomogram’s performance was evaluated using a variety of metrics. The Brier score was used to indicate the overall model performance, the area under the curve (AUC) of the receiver operating characteristics for model discriminating ability assessment, and a calibration plot for detecting the agreement between the observed and predicted probability of developing HIV infection (34). Additionally, accuracy, sensitivity, specificity, and predictive values were also calculated to assess the performance of the model (35). In order to choose the optimal probability cutoff point for risk stratification as high or low, the Youden’s index method was applied (36). An individualized risk stratification table was constructed based on the identified cutoff point to assign each woman to either the high- or low-risk group and to facilitate prevention interventions based on the evaluated risk of developing the HIV infection (37). The internal validity of the model was evaluated using the bootstrapping method (38). Bootstrapping provides stable estimates with low bias as compared to other methods of internal validation, such as the split sample (39). The difference between the apparent performance (performance before validation) and true performance (performance after validation) was calculated. The transparent reporting of a multivariable prediction model for an individual prognostic or diagnostic checklist was used to report the findings of the study (40).

**Figure 1:**
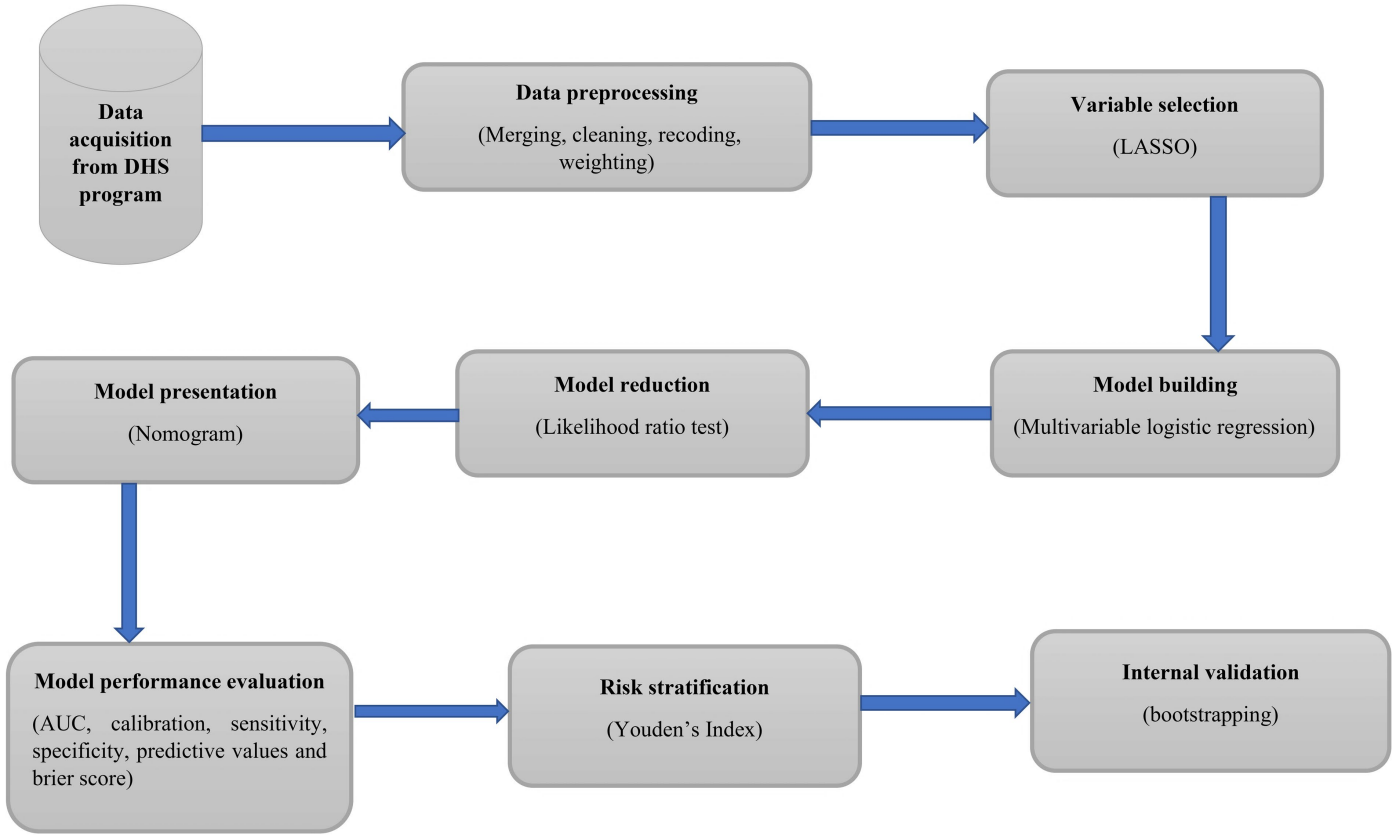
Workflow of a study to develop and validate an individualized risk prediction model for HIV infection among sexually active women in Ethiopia.

The overall modeling process is presented in Figure 1.

### Data collection and instruments

The 2016 EDHS data were collected through face-to-face, interviewer-administered questionnaires. Both women’s and bio-marker questionnaires, which include sociodemographic and economic factors, behavioral characteristics, and HIV-testing results were used for data collection. During HIV testing, all samples were tested with the Genscreen ULTRA Ag/Ab (Bio-Rad) enzyme-linked immunoassay (ELISA I). All samples that tested positive for ELISA I were subjected to a second ELISA (ELISA II) and the Bioelisa HIV 1 + 2 Ag/Ab combination (Biokit). In total, 5% of the samples that tested negative for ELISA I were also subjected to ELISA II, while the other 95% were recorded as negative. Concordant negative results on ELISA I and ELISA II were recorded as negative. If the results of ELISA I and ELISA II were discordant, the specimen was considered inconclusive. Concordant positive results on ELISA I and ELISA II were also subjected to a third confirmatory assay. Accordingly, if an individual had positive test results for both ELISA I and II, the final HIV test result was considered to be positive, if the confirmatory test (Inno-Lia) was positive. On the other hand, the final HIV test was taken as an inconclusive result for an individual who had positive test results for both LISA I and II if the Inno-Lia was a negative or indeterminate result (32).

### Ethical considerations

During the 2016 EDHS survey, all participants in the HIV test granted their consent for blood sample collection. Individuals who did not grant consent for participation in the HIV test were not included (32). In this study, we used publicly available data with no personal identifiers, which was provided by the DHS program. All methods were carried out in accordance with the relevant guidelines of the DHS program.

## Results

### Characteristics of participants

The mean (SD) age of participants was 31 (8.3) years, of which more than one-fifth of women were between 25 and 29 years old. Approximately 6 out of 10 women (58.9%) did not attend formal education. The majority (84.9%) of the participants were married/cohabiting during the survey. Approximately one-fourth (23.3%) of participants lived in households with the richest wealth index. More than three-fourths (78.2%) of women lived in male-headed households. Regarding age at first sex, 23.3% of women started sexual intercourse at or before 14 years of age. A total of 14.8 and 24.8% of sexually active women had multiple sexual partners in the last year prior to the survey and in their lifetime, respectively. The majority (80.6%) of women resided in urban areas (Table 1).

**Table 1:** Characteristics of study participants, EDHS 2016.

Variable	Category	Weighted frequency (*n* = 10,253)	Percentage (%)
Age	15–19	693.6	6.8
	20–24	1741.4	17.0
	25–29	2315.0	22.6
	30–34	1946.2	19.0
	35–39	1603.6	15.6
	40–44	1085.5	10.6
	45–49	867.4	8.5
Education	No	6035.5	58.9
	Primary	3013.1	29.4
	Secondary	731.5	7.1
	Higher	472.5	4.6
Marital status	Single	338.4	3.3
	Married/cohabiting	8702.5	84.9
	Widowed/divorced/separated	1211.7	11.8
Wealth index	Poorest	1918.3	18.7
	Poorer	1974.6	19.3
	Middle	2015.7	19.7
	Richer	1960.7	19.1
	Richest	2383.3	23.3
Sex of the household head	Male	8016.9	78.2
	Female	2235.7	21.8
Age at first sex (years)	≤14	2386.9	23.3
	15–17	4436.2	43.3
	≥18	3429.5	33.4
Multiple sexual partners in the last year	Yes	1514.7	14.8
	No	8738.0	85.2
Multiple sexual partners during lifetime	Yes	2441.3	23.8
	No	7811.3	76.2
Diagnosed STIs	Yes	28.7	0.3
	No	10223.9	99.7
Genital ulcers	Yes	208.0	2.0
	No	10044.6	98.0
Genital discharge	Yes	262.1	2.6
	No	9990.5	97.4
Residence	Urban	1994.2	19.4
	Rural	8258.4	80.6

### National and regional prevalence of HIV infection

The overall prevalence of HIV among sexually active women in Ethiopia was 1.5% (95% CI; 1.3, 1.8%). The lowest and highest prevalences were found in the Somali (0.1%) and Gambela (6.7%) regions, respectively (Table 2).

**Table 2:** National and regional prevalence of HIV infection among sexually active women in Ethiopia, EDHS 2016.

Region	Sampled women	Weighted HIV-positive frequency	Percentage of HIV (%)
Tigray	749.0	13.8	1.8
Afar	94.3	1.6	1.7
Amhara	2538.3	41.0	1.6
Oromia	3829.2	44.2	1.2
Somali	310.1	0.3	0.1
Benishangul Gumuz	109.5	2.1	1.9
SNNPR	2012.8	13.6	0.7
Gambela	31.4	2.1	6.7
Harari	26.0	1.1	4.2
Addis Ababa	494.5	32.2	6.5
Dire Dawa	57.5	2.6	4.5
Ethiopia (Total)	10,253	154.5	1.5

### Development of an individualized HIV risk prediction model

#### Predictor selection

A total of 4 out of the 13 predictor variables (STI, genital ulcer, genital discharge, and region) did not meet the chi-square assumption and were therefore not included in the LASSO regression model. The remaining nine variables were entered into LASSO regression, and non-zero coefficients were identified at a tuning parameter (lambda) of 0.0004 with a cross-validated mean deviance of 0.20 and an out-of-sample deviance ratio of 0.18 at the 46th iteration. All variables included in the LASSO had non-zero coefficients and were thus included in the multivariable model.

#### Multivariable mixed-effects logistic regression model

The ICC value was estimated to be 37.2% (95% CI: 28.9, 46.4). Therefore, to account for the variability explained by the clustering effect, variables with non-zero coefficients from the LASSO regression were exported into a mixed-effects multivariable binary logistic regression model. To achieve model parsimony, further model reduction was conducted by reducing insignificant variables (*p*-value >0.05) using the likelihood ratio test. Two variables, namely, household wealth index (*p*-value = 0.076) and multiple sexual partners in 1 year (*p*-value = 0.153), were removed. The full model (model with nine predictors) and the reduced model (model with seven predictors) were compared using the likelihood ratio test, which implies that no statistically significant difference was observed between the two models (likelihood ratio chi-square = 6.98 and *p*-value = 0.222). Finally, seven predictors were retained and used for nomogram development (Table 3).

**Table 3:** Adjusted odds ratios from multivariable mixed-effects logistic regression model, EDHS 2016.

Variables	Category	AOR (95% CI)	β-coefficients	*p*-value
Age group	15–19	Ref		
	20–24	0.76 (0.30, 1.94)	−0.28	0.562
	25–29	2.15 (0.93, 4.97)	0.77	0.073
	30–34	4.21 (1.85, 9.59)	1.44	0.001
	35–39	3.67 (1.60, 8.41)	1.30	0.002
	40–44	3.16 (1.34, 7.43)	1.15	0.008
	45–49	2.64 (1.08, 6.44)	0.97	0.032
Educational status	No	Ref		
	Primary	2.50 (1.77, 3.53)	0.92	< 0.001
	Secondary	2.39 (1.53, 3.73)	0.87	< 0.001
	Higher	1.21 (0.67, 2.19)	0.19	0.535
Marital status	Single	Ref		
	Married/cohabiting	1.21 (0.67, 2.18)	0.19	0.525
	Widowed/divorced/separated	2.39 (1.33, 4.32)	0.87	0.004
Sex of the household head	Male	Ref		
	Female	2.05 (1.48, 2.85)	0.72	< 0.001
Age at first sex	≤14	1.52 (1.05, 2.21)	0.42	0.026
	15–17	1.21 (0.89, 1.68)	0.20	0.224
	≥18	Ref		
Multiple sexual partners during lifetime	Yes	2.61 (1.98, 3.45)	0.96	< 0.001
	No	Ref		
Residence	Urban	4.29 (2.97, 6.20)	1.46	< 0.001
	Rural	Ref		
Intercept		0.0006 (0.0002, 0.0018)	−7.30	< 0.001

The probability of developing HIV infection among sexually active women was predicted using the following regression formulas described in Equations 1, 2.


(1) $$\begin{array}{l} \mathrm{Linear predictor of the model}\;\left(\mathrm{lp}\right)=-7.30+1.44\\ {}*\mathrm{age}\;\left(30-34\right)+1.30*\mathrm{age}\;\left(35-39\right)+1.15\\ {}*\mathrm{age}\;\left(40-44\right)+0.97*\mathrm{age}\;\left(45-49\right)+0.92\\ {}*\mathrm{education}\;\left(\mathrm{primary}\right)+0.87*\mathrm{education}\;\left(\mathrm{secondary}\right)+0.87\\ {}*\mathrm{marital status}\;\left(\mathrm{separated}/\mathrm{divorced}/\mathrm{widowed}\right)+0.72\\ {}*\mathrm{sex}\;\mathrm{of household head}\;\left(\mathrm{female}\right)+0.42\\ {}*\mathrm{age}\;\mathrm{at}\;\mathrm{first}\;\mathrm{sex}\;\left(\leq 14\;\mathrm{years}\right)+0.96\\ {}*\mathrm{lifetime multiple sexual partners}\;\left(\mathrm{yes}\right)+1.46\\ {}*\mathrm{residence}\;\left(\mathrm{urban}\right)\ldots \end{array}$$

Consequently, the probability of being HIV^+^ is given by the following equation:


(2) $$\mathrm{HIV}+=\frac{\exp^{lp}}{1+\exp^{lp}}$$

### Nomogram for predicting individualized HIV infection risk

**Figure 2:**
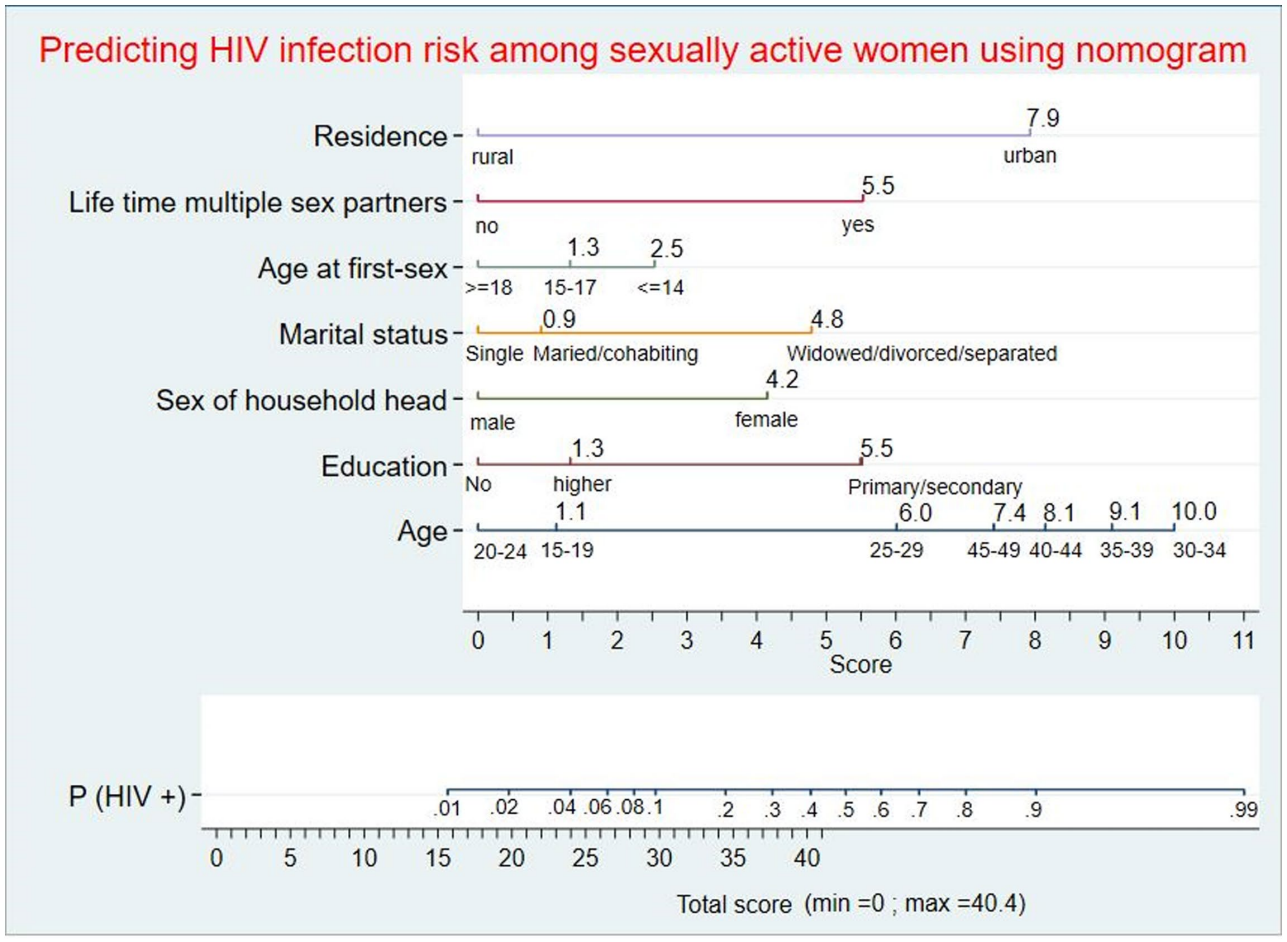
Nomogram for predicting individualized risk of HIV infection among sexually active women in Ethiopia. An individual with a total score of 21 will correspond to a 0.0281 probability of developing HIV infection; women with a ≥ 0.0281 probability will be classified as high-risk, whereas women with a < 0.0281 probability will be classified as low-risk during nomogram application.

For ease of clinical applicability, the nomogram was developed using the seven predictors from the reduced model. Scores corresponding to each predictor were generated from the nomogram division table automatically. Based on the total score, the individualized probability of developing HIV infection could be calculated easily using the nomogram (Figure 2).

### Performance of the nomogram

**Figure 3:**
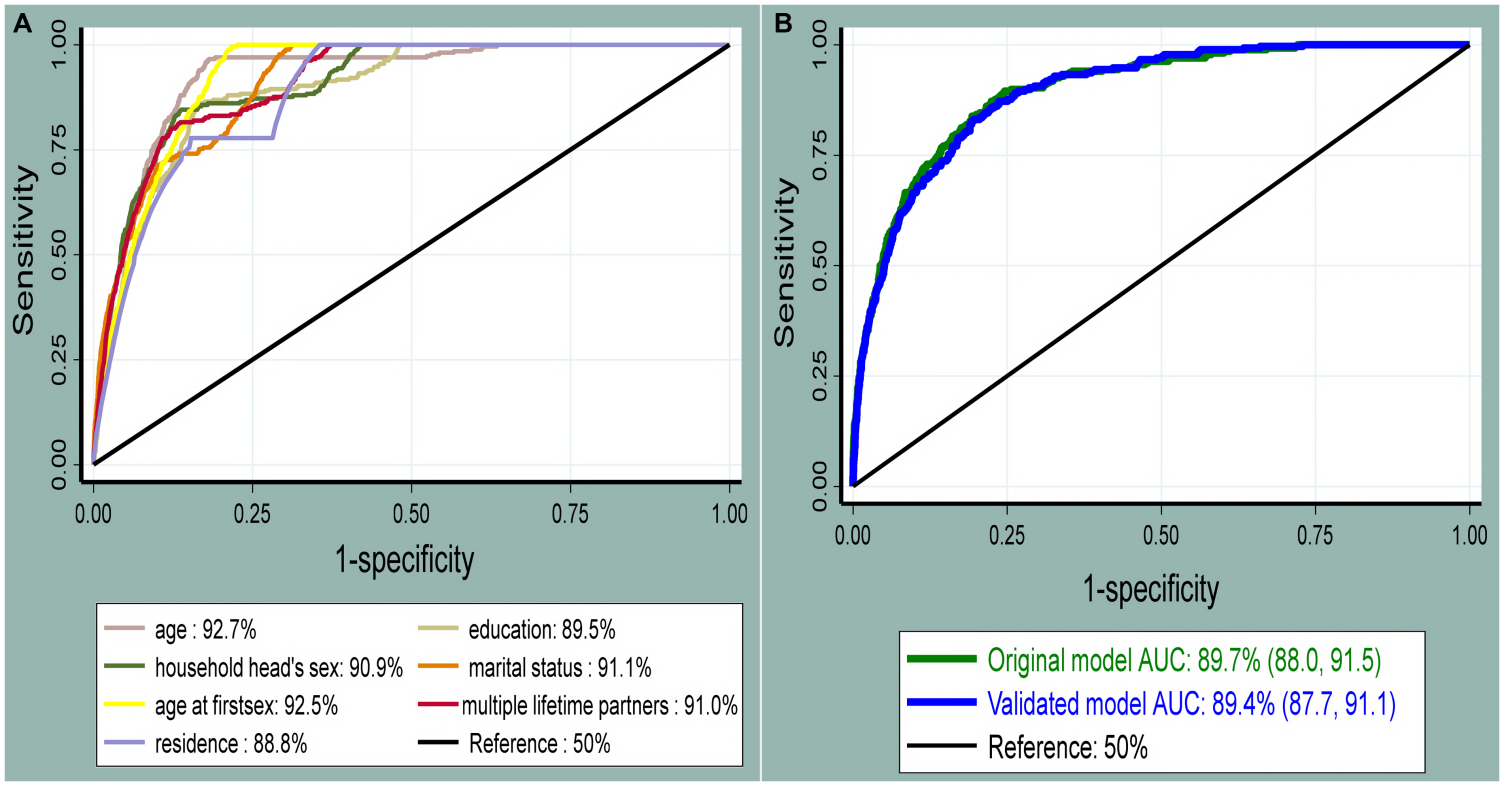
The receiver operating characteristic curve shows the performance of each predictor **(A)** and the original and internally validated model **(B)**. The diagonal black line represents a model that discriminates by chance (AUC = 50%); the x-axis shows the proportion of individuals without HIV infection who were incorrectly classified as having HIV (false-positive rate), and the y-axis shows the proportion of individuals with HIV who were correctly classified as having HIV infection (true-positive rate).

**Figure 4:**
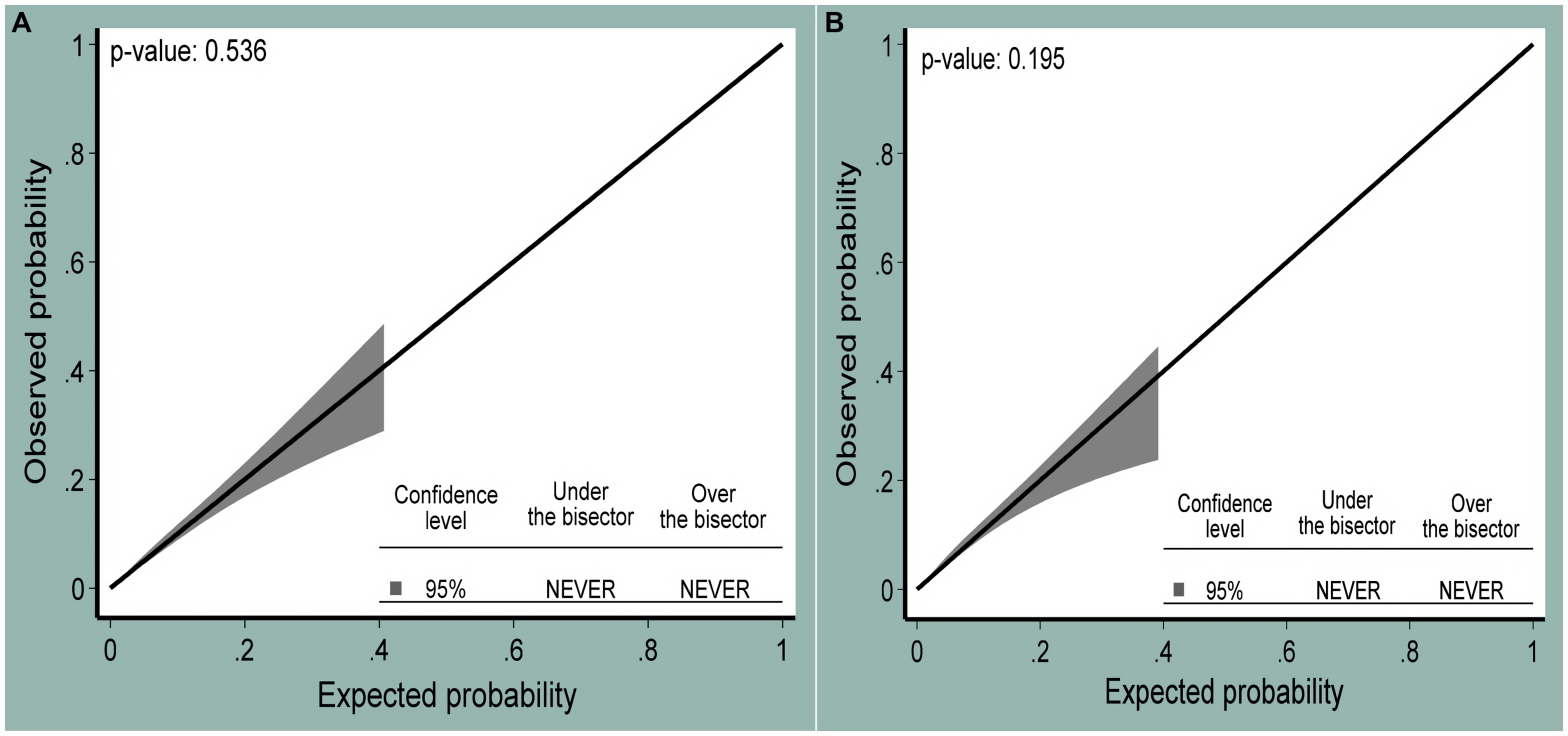
The calibration plot showing the agreement between observed (y-axis) and expected (x-axis) probabilities of being HIV positive at 95% confidence levels for the original **(A)** and internally validated models **(B)**.

The role of each predictor for the overall AUC was presented (Figure 3A). The AUC of the nomogram for individualized HIV risk prediction was 89.7% (95% CI: 88.0, 91.5) (Figure 3B). When additional performance measures were assessed, the model had a sensitivity of 74.1% (95% CI: 68.4, 79.2), specificity of 80.9% (95% CI: 80.2, 81.7), PPV of 8.7% (95% CI: 7.6, 9.9), NPV of 99.2% (95% CI: 99.0, 99.4), and an accuracy of 80.8% (95% CI: 80.0, 81.5) to identify individuals at risk for HIV at the 0.0281 probability cutoff point identified by Youden’s index (max J = 0.55). The calibration test had a *p*-value of 0.536, indicating no significant difference between the observed probability of developing HIV and the expected probability of developing HIV (the calibration plot includes the bisector) (Figure 4A). The Brier score of the model was 0.02.

### Individualized HIV infection risk stratification table

The risk stratification table was constructed using Youden’s index (max J = 0.55), and the corresponding probability cutoff point with this index was 0.0281. The risk was dichotomized into low- (<0.0281) and high-risk (≥0.0281) groups. Of the total participants, 1,249 (12.2%) were in the high-risk group. Of all HIV infections, the majority (73.4%) of HIV-positive individuals were from the high-risk category. The prevalence of HIV was 0.5 and 9.1% in low-risk and high-risk groups, respectively (Table 4).

**Table 4:** Risk stratification table based on the probability of HIV infection identified by the nomogram.

Risk category (probability)	Nomogram	
	Frequency	Frequency (prevalence) of HIV^+^
Low risk (*p* < 0.0281)	9,004 (87.8%)	41 (0.5%)
High risk (*p* ≥ 0.0281)	1,249 (12.2%)	113 (9.1%)
Total	10,253 (100%)	154 (1.5%)

### Internal validation

The developed nomogram was internally validated by bootstrapping using 1,000 bootstrap samples with replacement to determine the degree of overfitting (i.e., models performing better in the development sample than in the new sample after bootstrapping). The AUC of the internally validated model was 89.4% (95% CI: 87.7, 91.1) (Figure 3B). Additional model performances were also evaluated after internal validation. The model’s sensitivity was 72.9% (95% CI: 67.2, 78.2), the specificity was 80.1% (95% CI: 79.3, 80.9), the PPV was 8.2% (95% CI: 7.1, 9.4), the NPV was 99.2% (95% CI: 99.0, 99.4), and the accuracy was 80.0% (95% CI: 79.2, 80.7) at the 0.0281 probability cutoff point identified by a Youden’s index of 0.53. The calibration curve p-value was 0.195, which indicated the presence of agreement between the observed and predicted probability of mortality across all probability thresholds (bisector) (Figure 4B). The optimism coefficient and Brier score were 0.003 and 0.02, respectively.

## Discussion

This study aimed to develop an individualized risk prediction tool that helps predict the probability of developing HIV infection for a sexually active woman. Sexually active women are a vulnerable population group for HIV. Women are more prone to HIV infection than men due to biological differences and gender inequalities (29). Developing a risk prediction model for this highly vulnerable group of the population plays a vital role in the prevention of HIV infection and subsequent reduction of HIV prevalence. This will make a great contribution to the global target of ending HIV as a public health threat. Prediction models direct the decision-making process in the choice of interventions through the estimation of individualized risk with the aim of achieving better outcomes (30, 31, 41).

The nomogram risk prediction model was constructed using multivariable logistic regression analysis, considering the clustering effect. Seven predictors were selected for model development after passing through LASSO regression and model reduction. The developed model was assessed for internal validity using a bootstrapping technique. The developed nomogram could assist health professionals in their delivery of HIV prevention services. Nomograms are easy-to-use graphical tools during the application (42).

The odds of having HIV infection among sexually active women were higher among older age groups compared with women aged 15–19 years. This finding is supported by studies from Nigeria (16) and Kenya (18). The positive relationship between increased age and HIV infection might be because older women have relatively more repeated exposure to risk factors for HIV than younger women. In Ethiopia, higher HIV prevalence is reported in older age groups (43).

Those with primary and secondary education had higher odds of HIV infection as compared to women who had no education. Previous studies have found that better levels of education are related to an increased risk of HIV infection due to the fact that more educated women are more likely to be mobile and have more sexual partner networks (17). Being widowed, divorced, or separated was found to be significantly associated with higher odds of HIV infection. This finding is supported by studies from Nigeria (16) and Zambia (44). Based on a recently conducted population survey in the SSA, widowed and divorced women are more likely to engage in high-risk behaviors for substance abuse which may increase the probability of engaging in risky sexual behaviors (45). The sex of the household head was also significantly associated with HIV infection. Sexually active women living in female-headed households have higher odds of HIV infection as compared to sexually active women living in male-headed households. This finding is supported by previous studies from Ethiopia (21) and SSA (46). In Ethiopia, men are usually heads of household. Nevertheless, when women are heads of household, it is a sign that the woman’s marital status is unmarried, divorced, or widowed. The probability of women engaging in adopting high-risk sexual activities, including multiple partners, engagement, in paid sex, and sexual harassment. Women who started having sex at or before the age of 14 were more likely to contract HIV. This finding is supported by evidence from Tanzania (22), Zimbabwe (47), and Rwanda (48). This may be because those who start having sex early are more likely to have multiple sexual partners in their lifetime. Having multiple sexual partners in life increases the odds of HIV infection. This is consistent with findings from other studies (17, 18). Sexually active women residing in urban areas had higher odds of HIV infection compared to sexually active women living in rural areas. This is supported by another study from Ethiopia (49). In Ethiopia, urban areas had a higher prevalence of HIV infection, which may be a possible reason for the higher transmission of HIV among these residents (43).

The optimal combination of variables to predict HIV infection using a nomogram was age group, education, marital status, sex of the household head, age at first sex, multiple sexual partners during the lifetime, and place of residence. The combined performance of these women’s sociodemographic characteristics and sexual behavior resulted in a prediction nomogram with an AUC of 89.7% (95% CI: 88.0, 91.5), which was well-calibrated (*p*-value = 0.536). This HIV risk prediction model had very good discriminatory power, according to the prediction model performance classification (50). The performance of this nomogram was better than that of a study conducted in Chicago that developed a predictive model for identifying women at risk for HIV, which had an AUC of 74% (95%CI: 67, 81). Variables included were as follows: STIs, substance use, hepatitis C, pregnancy, race, ethnicity, age group, healthcare site, and number of medical encounters (51). In addition, the performance of the developed nomogram in this study was better than a prediction risk score for HIV among adolescent girls and young women in South Africa that was conducted to identify those in need of HIV pre-exposure prophylaxis based on their elevated risk, which had an AUC of 78% in the development and 76% in the validation data, respectively (52). Variables included in a study conducted in South Africa were age, age at first sex, relationship status, education, socioeconomic status, number of sexual partners, any STI, HIV-positive partner, condom use at last sex, ever engaged in transactional sex, parent dead, ever pregnant, and ever raped. The variation in model performance between the two studies might be due to differences in the strength of the included predictors for model development (53).

Based on Youden’s index, an optimal probability cutoff point of 0.0281 was identified and resulted in better model performance (including discrimination, sensitivity, specificity, and predictive values) compared to other cutoff probability points. Using this probability, the cutoff point for HIV risk prediction the model performance would have good benefits in a risk-stratified intervention. This means that women with a probability of at least 0.0281 should be screened and linked to HIV prevention services. Intentional efforts are required to recruit such high-risk sexually active women for prevention services to reduce their likelihood of serostatus conversion. However, the optimal probability cutoff point identified through Youden’s index does not replace routine HIV screening services offered in clinical care settings. The HIV risk prediction nomogram is meant to support healthcare professionals in stratifying sexually active women based on their individualized risk for HIV infection and providing services according to their risk strata. These findings inform users of the developed nomogram that HIV services for sexually active women should particularly target those with elevated risk to ensure HIV prevention interventions are cost-effective and impactful (52, 54).

The nomogram was validated internally using the bootstrap resampling technique. The discriminatory performance of the validation sample was 89.4% (95% CI: 87.7, 91.1), and regarding calibration, it was well calibrated (p-value = 0.195). The optimism coefficient was 0.003, indicating that the nomogram is less likely to be sample-dependent. Hence, the nomogram can be used for risk prediction among sexually active women in Ethiopia.

### Policy implications and applications

Our nomogram has policy implications and applications for HIV/AIDS prevention. First, the developed nomogram may be used by clinicians and public health experts to enhance counseling and health education for sexually active women in highly HIV-prevalent areas of Ethiopia, which is an important strategy to improve HIV prevention services, including screening and pre-exposure prophylaxis. Second, a nomogram may also be useful to monitor changes in risk over time if the sexually active woman’s risk is changing from low to high or high to low. Third, this nomogram may be used to allocate more resources to areas that have a large number of high-risk sexually active women; hence, equitable resource allocation will occur.

### Strengths and limitations of the study

The strengths of this study are as follows: first, we use nationally representative data, which maintains its generalizability for all sexually active women in Ethiopia. Second, to our knowledge, this is the first nomogram developed for the prediction of HIV in the country. Third, the small optimism coefficient identified in the internal validation process indicates a less likely overfitting of the nomogram, and hence it can predict HIV when applied to an independent set of samples with very good performance. However, the limitations of this study are as follows: first, limited applicability in other countries due to a lack of external validation, thus, nomogram applicability is subjected to external validation for other countries. Second, the use of prevalence data instead of incidence data to develop a risk prediction model for sexually active women may affect the nomogram’s performance in predicting new HIV infections.

## Conclusions and recommendations

Our nomogram has shown very good discrimination and good calibration to identify sexually active women with a high risk of developing HIV infection. This model directs high-risk women to be targeted for intensive HIV prevention interventions at both health facilities and community levels. Therefore, we recommend the use of this nomogram by health professionals to guide their decision-making process when providing HIV prevention services. Researchers should conduct a feasibility study on the use of the nomogram in clinical and community settings to assess its user-friendliness and accuracy in identifying HIV in high-risk sexually active women. External validation should be conducted before the application of the model in other HIV-epidemic countries.

## Data availability statement

The original contributions presented in the study are included in the article/supplementary material, further inquiries can be directed to the corresponding author.

## Ethics statement

Ethical considerations during EDHS 2016 data collection were approved by the ICF Institutional Review Board (IRB). Moreover, EDHS protocols were reviewed by the ICF IRB and by an IRB in Ethiopia during the data collection period. Before each interview or HIV test was conducted, an informed consent statement was read to the respondent, who might accept or decline to participate. The informed consent maintained voluntary participation and autonomy while strictly maintaining confidentiality and privacy. Individuals who did not grant consent for participation in HIV testing were not included (30). In this study, we used a publicly available data provided by the DHS program, with no personal identifiers. All methods were carried out in accordance with the relevant guidelines of the DHS program after we received a letter of permission from the Institutional Review Board of the DHS program. The studies were conducted in accordance with local legislation and institutional requirements. Written informed consent for participation was not required from the participants or the participants’ legal guardians/next of kin in accordance with national legislation and institutional requirements.

## Author contributions

TT: Conceptualization, Data curation, Formal analysis, Investigation, Methodology, Project administration, Resources, Software, Supervision, Validation, Visualization, Writing – original draft, Writing – review & editing. TY: Formal analysis, Methodology, Software, Validation, Visualization, Writing – original draft, Writing – review & editing. MA: Formal analysis, Investigation, Methodology, Software, Supervision, Validation, Visualization, Writing – review & editing. GY: Methodology, Software, Validation, Visualization, Writing – original draft. SW: Formal analysis, Investigation, Methodology, Software, Visualization, Writing – original draft. BF: Formal analysis, Software, Validation, Writing – original draft, Writing – review & editing. ND: Formal analysis, Methodology, Software, Validation, Visualization, Writing – original draft, Writing – review & editing.
